# Ablative Techniques for Lung Metastases: Patient Selection and Outcomes Following Treatment with Stereotactic Radiotherapy or Radiofrequency Ablation

**DOI:** 10.3390/curroncol32060303

**Published:** 2025-05-25

**Authors:** Jennifer W. S. Pang, Daniel Tong, Nicos Fotiadis, Laura Satchwell, Zayn Rajan, Mohammad Emarah, Helen Taylor, Usman Bashir, Derfel Ap Dafydd, James McCall, David Cunningham, Merina Ahmed

**Affiliations:** 1 Maidstone and Tunbridge Wells NHS Trust, Maidstone Hospital, Hermitage Lane, Maidstone ME16 9QQ, UK; 2 Royal Marsden NHS Foundation Trust, Downs Rd, Sutton SM2 5PT, UK; 3 Barts Health NHS Trust, Whitechapel Rd, London E1 1BB, UK

**Keywords:** Stereotactic radiotherapy, radiofrequency ablation, lung metastasis, outcome

## Abstract

Stereotactic radiotherapy (SBRT) and radiofrequency ablation (RFA) are common ablative techniques for lung metastases. A retrospective review of all patients treated with either modality at a single institution between 2011 and 2019 was conducted. Baseline characteristics and outcomes were compared. Local and distant progression, and overall survival were estimated using the Kaplan–Meier method. Univariable analysis was carried out using Cox regression; this was followed by multivariable modelling. In total, 106 patients treated with RFA and 70 treated with SBRT were identified. Tumours treated with SBRT were larger (median size 18 mm vs. 11 mm) and primarily oligometastatic (91.4% vs. 20%). Median progression-free survival (PFS) was 12.5 months for SBRT and 7.9 months for RFA (*p* = 0.009). Median OS was similar (*p* = 0.66). In multivariable analysis, lesion size > 20 mm was predictive of adverse local PFS (*p* = 0.001), PFS (*p* = 0.0034) and OS (*p* = 0.001). A statistically significant interaction effect suggested that RFA was associated with better local PFS within colorectal primary patients (*p* = 0.035). This study highlights differences in patient selection and outcomes for RFA or SBRT in the treatment of lung metastases at our institution. Future studies for SBRT should focus on the optimum dose schedules required for different histologies. For less-radiosensitive tumours, RFA may offer a superior alternative where dose-escalated SBRT is not possible.

## 1. Introduction

Distant metastatic disease has historically been treated as incurable disease; however, the concept of an oligometastatic state with the potential for long-term disease control is now widely accepted. Surgical and non-surgical techniques are used with curative intent for treatment of oligometastases throughout the body, with a growing body of evidence for its survival benefit. Focusing specifically on lung metastases, the International Registry of Lung Metastases [1] (IRLM) was a registry study established in 1991 which suggested significant survival benefits gained from resection. PulMiCC was a two-arm randomized controlled trial in colorectal cancer patients which looked at whether adding metastasectomy to active monitoring improved survival [2]. This study was underpowered due to poor recruitment, and failed to show survival benefit, with 95% confidence intervals of overall survival crossing 1. Metastasectomy continues to be performed in routine clinical practice. For patients who are not suitable candidates for surgery, ablative techniques are widely used. However, it is not known which, if any, of these techniques are superior to another, or whether we should be selecting certain patient groups for a particular treatment modality, thereby individualising patient care.

The rationale for the use of non-surgical ablative techniques is that the existing data demonstrate similar rates of efficacy and long-term survival to those of surgically treated patients in the IRLM [3,4]. Ablation of lung oligometastases with stereotactic ablative body radiotherapy (SBRT) is a potential life-prolonging treatment, as supported by the survival benefits reported in the long-term follow-up of two randomized phase II trials [5,6]. Radiofrequency ablation (RFA), microwave ablation (MWA) and cryoablation are also widely used, with the largest body of evidence supporting SBRT and RFA [5,6,7,8,9,10,11,12,13,14,15,16,17,18,19,20,21,22]. The existing literature largely consists of outcomes following treatment with a single modality such as SBRT or RFA. Comparisons of ablative techniques in the literature are limited to tumour site-specific case series [23].

With the current lack of randomised evidence to direct treatment selection, we aim to identify disease or patient-related factors which affect the choice of local ablative treatment. In this retrospective study, we present the baseline characteristics of patients treated with RFA or SBRT for lung metastases at our institution. We report long-term outcomes with each treatment modality and review any potential correlation between baseline characteristics and any observed differences in outcomes.

## 2. Materials and Methods

### 2.1. Patients

We included all patients who underwent RFA or SBRT for treatment of a lung metastasis between November 2011 and December 2019 at our institution, following Institutional Research and Development committee approval. As the primary objective assessed was local progression-free survival (LPFS), patients required at least one follow-up CT scan at 3 months after treatment to be included.

The following data were collected retrospectively using electronic patient records: age, gender, primary diagnosis, date of treatment, number of lesions treated simultaneously, size of metastases, acute (within 3 months) and late (≥3 months post treatment) toxicities (CTCAE v4.0), ECOG performance status, oligometastatic status at the time of local intervention (defined as up to 3 sites of extracranial metastases), time to local and distant progression, site of distant progression, date of death, and any systemic treatment preceding or following the current therapy.

“Local treatment” included surgery and non-surgical techniques (either RFA or SBRT). Synchronous extrapulmonary disease was recorded as “amenable to local treatment” if the disease was treated prior to the lung treatment, or if local treatment was planned to take place following the lung treatment. Any systemic treatment that was given prior to or following the RFA or SBRT episode was recorded, regardless of the time interval from the episode being evaluated. Biologically Effective Dose (BED) was calculated with the following formula: BED = Total dose × (1 + (Dose per fraction/alpha-beta ratio)).

### 2.2. Imaging Review

All imaging was reviewed by two independent consultant radiologists specifically for the purpose of this evaluation to assess tumour size, site and local control. Local progression was defined for RFA patients as per Lencioni et al. as being any increase in the ablation zone of the baseline CT carried out 4–6 weeks post ablation, any new enhancement of the ablation zone, or an increase of more than 20% in the treated lesions (using modified RECIST criteria) [24]. For SBRT patients, the criteria as defined by Huang et al. were used [25]. PFS was defined as the time from the start of the initial treatment to the date of either local or distant progression, whichever was earlier. Distant progression was defined as progression of any disease outside of the lesion being treated.

### 2.3. Statistical Analysis

Descriptive statistics were generated for each of the baseline characteristics, adverse events, and other outcome data, and were presented both overall and by treatment cohort. The categorical groups were compared using the Chi-squared test for independence, applying a 95% confidence interval. Numerical groups were compared using a non-parametric Wilcoxon rank-sum test due to skewed data.

Local progression, distant progression and overall survival were estimated using the Kaplan–Meier method, and the log-rank test was used for comparison by treatment cohort, with *p* ≤ 0.05 being considered significant. Patients who did not experience any event of interest were censored at their last follow-up date. Subgroups of interest (primary diagnosis (colorectal vs. others) and largest treated lesion size (≤20 mm vs. >20 mm)) were also examined using these methods. Local progression-free survival was defined as being progression-free in the treatment field, and PFS as being progression-free in local and distant sites.

Univariable models were first constructed with treatment cohort as the only covariate. Subsequent univariable models looked at each of the other prognostic factors separately, adjusting for treatment cohort. Prognostic factors of interest were as follows: lesion size (≤20 mm vs. >20 mm), previous systemic anti-cancer therapy (SACT), subsequent SACT, oligometastatic disease, >1 lesion treated simultaneously, colorectal vs. non-colorectal primary. Following this, a multivariable model was constructed using a backward-elimination stepwise procedure, with a *p*-value cutoff of 0.05. All the variables in univariable analysis were considered for the final multivariable model, with treatment cohort, lesion size and colorectal primary being forced into the model irrespective of statistical significance.

The final model after this model-fitting process was presented with adjusted hazard ratios and the *p*-value associated with each term in the model. Statistically significant variables at the 5% level were highlighted. Stata 18 software was used to perform all statistical analyses (Version 18, Chicago, IL, USA).

## 3. Results

### 3.1. Baseline Demographics

We retrospectively identified 106 patients who received RFA and 70 patients who were treated with SBRT. The baseline characteristics and differences are highlighted in Table 1. The SBRT cohort contained mostly oligometastatic patients (91.4%), whereas 20% of those treated with RFA were oligometastatic (Table 1). The SBRT cohort had larger treated tumours (median size 18 mm vs. 11 mm) and more males in the cohort (63.4% vs. 50%). The RFA cohort was younger (median age 65 vs. 70.5) and more likely to have more than one lesion treated simultaneously (27.4% vs. 12.9%). The RFA cohort also received more systemic therapy, both before (76% vs. 55.7%) and after local ablative treatment (72% vs. 51.4%).

**Figure 1:**
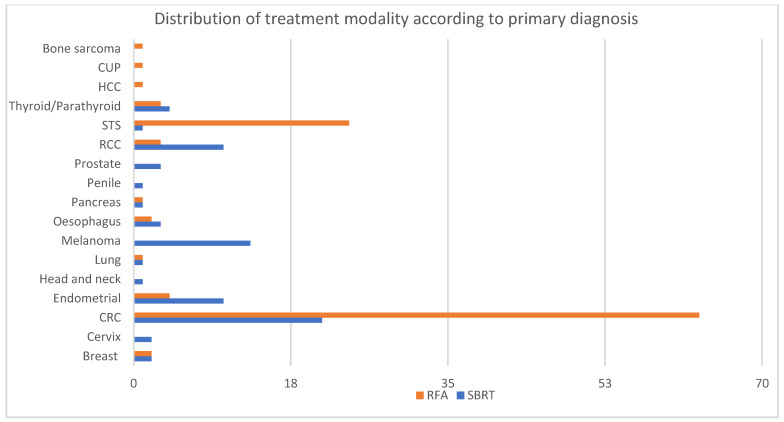
Distribution of primary diagnoses treated by SBRT and RFA. RCC—renal cell carcinoma; STS—soft-tissue sarcoma; HCC—hepatocellular carcinoma; CUP—carcinoma of unknown primary; CRC—colorectal cancer.

The most commonly treated primaries were patients with colorectal cancer (CRC) and soft tissue sarcoma (STS) primaries (47.7% and 14.2%, respectively), followed by endometrial cancer, melanoma and renal cell carcinoma (RCC) (8%, 7.4% and 7.4%, respectively). The distribution of primary diagnoses differed between the two treatment modalities (Figure 1). The majority of patients with melanoma (100%), RCC (77%) and endometrial cancer (71%) were treated with SBRT. On the other hand, patients with STS (96%) and CRC (75%) were preferentially treated with RFA.

### 3.2. Adverse Events

In the SBRT group, 51.4% experienced grade 1–2 acute toxicity, with fatigue being the commonest form (37.1%), followed by cough (12.8%). One patient (2.9%) experienced acute toxicity of grade 3 or above. In terms of late effects, eight patients (11.4%) experienced grade 1–2 late toxicity, with three patients developing grade 1–2 pneumonitis. One patient (2.9%) experienced grade 3 pneumonitis four months following SBRT. This patient had been treated with Cyberknife, 54 Gy in three fractions. No other late toxicities of grade 3 or above were recorded in the SBRT group.

Patients receiving RFA were routinely observed overnight following the procedure, with chest X-ray being performed four hours post-procedure. Among those who received RFA, 49% of patients experienced grade 1–2 acute toxicity, with grade 1–2 pneumothorax being the most common complication (44.3% of the patients). None of these patients required hospitalization. Eight patients (7.5%) experienced acute toxicity of grade 3 or above. Four patients had grade 3 pneumothorax, two patients had a grade 4 chest infection, one patient had haemothorax, and one patient had grade 3 pericardial puncture. One patient had a contained air-embolism with air in the pulmonary veins and left atrium but no systemic embolism. This last patient developed a chronic necrotizing pneumonia and aspergillosis at the site of the ablation zone. Only one patient experienced late toxicity; this patient had grade 1 dyspnoea.

#### 3.2.1. Local Control by Primary Site

Local control (LC) was similar with both treatment modalities in breast, endometrial and RCC (Table S1). Better LC was observed in colon and rectal cancer patients treated with RFA, compared with SBRT (LC 89.7% vs. 64.2% and 96.2% vs. 71.4%, respectively).

#### 3.2.2. Local Control with SBRT by Biologically Effective Dose (BED)

The most common dose regimen used was 60 Gy in eight fractions (36.1%), followed by 55 Gy in five, 54 Gy in three, and 50 Gy in five (27.8%, 20.8 and 6.9%, respectively). As biologically effective doses vary widely depending on the primary site being treated, the radiotherapy dose delivered was converted to biologically effective doses with the following alpha–beta ratios: 1.5 for prostate, 2 for adenoid cystic, 2.5 for melanoma, 3 for RCC and thyroid papillary carcinoma, 4 for breast and sarcoma, and 10 for colorectal, oesophageal, cervical, endometrial and pancreatic cancer (Table S2).

**Figure 2:**
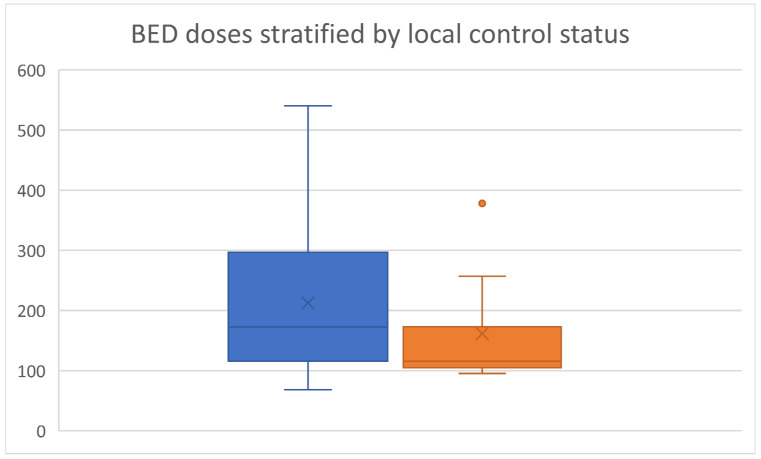
Box-and-whisker plot of BED doses in SBRT cohort stratified by local control status (left, in blue: local control; right, in orange: local failure).

Median BED dose prescribed was 151.2 Gy (range = 68–540 Gy, interquartile range (IQR) = 105–256.7 Gy). BED doses in those with local control and local failure are visually presented as a box-and-whisker plot in Figure 2. Among those with lack of local failure, the median BED dose prescribed was 172.5 Gy (IQR = 115.5–297 Gy. The median dose received by patients who experienced local failure was lower, at 115.5 Gy (IQR = 105–172.8 Gy), although the IQR values overlapped. The median BED dose prescribed to colorectal lung metastases was 105 Gy (IQR = 105–115 Gy).

### 3.3. Progression-Free Survival and Overall Survival

#### 3.3.1. Local PFS

**Figure 3:**
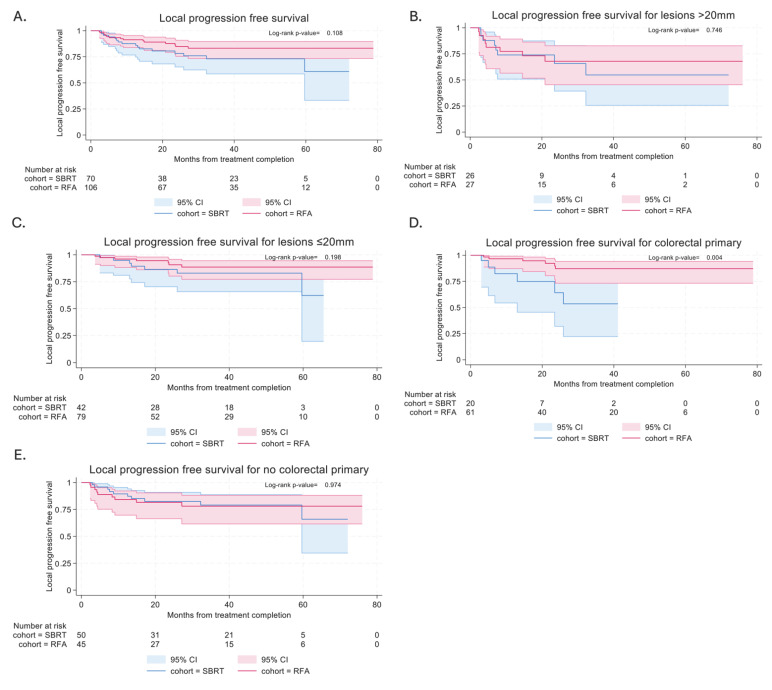
Local PFS after ablative treatment for lung metastasis. (**A**) In all patients treated with SBRT and RFA. (**B**) In lesions > 20 mm. (**C**) In lesions ≤ 20 mm. (**D**) In patients with colorectal primary. (**E**) In patients with non-colorectal primary.

Local PFS was similar in both SBRT and RFA cohorts, with the median time to local progression not being reached in either cohort (Figure 3A). In the SBRT cohort, local PFS at 6 and 12 months was 94.0% (95% CI: 84.7–97.7%) and 87.6% (95% CI: 76.7–93.6%), respectively. In the RFA cohort, local PFS at 6 and 12 months was 93.3% (95% CI: 86.5–96.8%) and 91.3% (95% CI: 83.9–95.4%), respectively.

Subgroup analysis of lesions stratified by size (≤20 mm vs. >20 mm) showed similar LPFS rates for both techniques in both smaller (*p* = 0.198) and larger lesions (*p* = 0.746) (Figure 3B,C). In view of the difference in local control between colorectal patients treated with RFA and SBRT, subgroup analysis was also performed. RFA was associated with better LPFS in colorectal patients (*p* = 0.004), and this effect was not maintained in non-colorectal patients (*p* = 0.974) (Figure 3D,E).

#### 3.3.2. Progression-Free Survival

**Figure 4:**
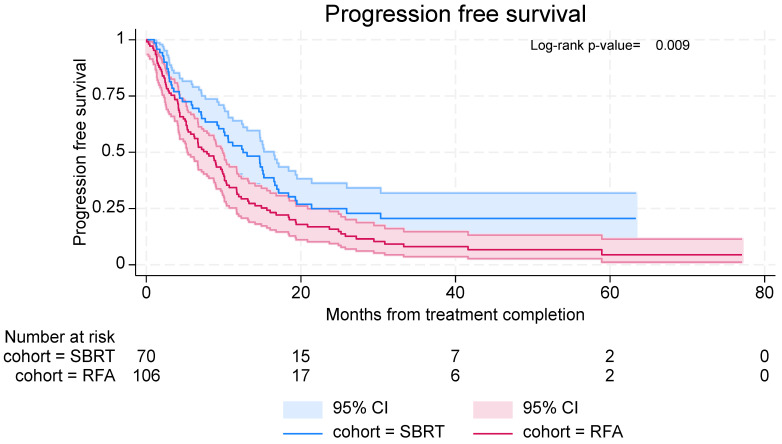
PFS in patients with lung metastases treated with SBRT and RFA.

Times to distant or local progression were longer in the SBRT cohort, with median time to progression being 12.5 months (95% CI: 9.2–16.5) in the SBRT cohort, compared with 7.9 months (95% CI: 5.3–9.9) in the RFA cohort (Figure 4). In the SBRT cohort, 6-month and 12-month PFS rates were 69.5% (95% CI: 57.1–79.0%) and 52.9% (95% CI: 40.3–63.9%), respectively. In the RFA cohort, 6-month and 12-month PFS rates were 58.0% (95% CI: 48.0–66.8%) and 30.3% (95% CI: 21.7–39.3%), respectively.

#### 3.3.3. Overall Survival

**Figure 5:**
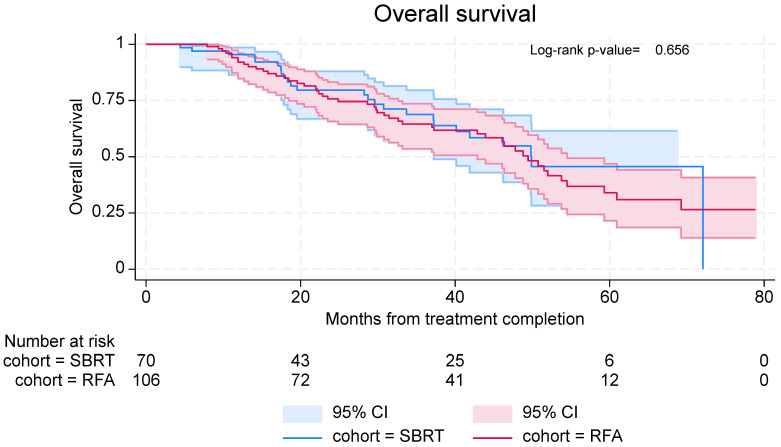
Overall survival in patients with lung metastases treated with SBRT and RFA.

Overall survival was similar in both groups, with median OS being 49.9 months (95% CI: 37.2—not reached) in the SBRT group and 49.4 months (95% CI: 42.93–54.5) in the RFA group (Figure 5). In the SBRT cohort, 6-month and 12-month OS rates were 97.0% (95% CI: 88.4–99.2%) and 95.4% (95% CI: 86.4–98.5%), respectively. In the RFA cohort, 6-month and 12-month OS rates were 100% and 92.1% (95% CI: 84.8–96.0%), respectively.

#### 3.3.4. Univariable and Multivariable Analysis

In both univariable and multivariable analysis, two factors were potentially associated with adverse survival outcomes, independent of ablative treatment modality: (i) lesions >20 mm were associated with adverse local PFS (HR 3.28 (95% CI: 1.59–6.78)), PFS (HR 1.47 (95%CI: 1.03–2.11)) and OS (HR 2.22 (95% CI: 1.38–3.58)), and (ii) those who did not require SACT following treatment correlated with longer PFS (HR 0.48 (95% CI: 0.32–0.70)) (Table S3). SBRT was associated with better PFS but was not associated with any difference in local PFS or OS.This difference was not maintained in the multivariable analysis, however.

#### 3.3.5. Cox Regression with Interaction Effects

Among patients with colorectal primaries, treatment with RFA was associated with better local control than SBRT (HR 0.21 (95% CI: 0.05–0.90)), *p* = 0.035. Lesion size >20 mm maintained statistical significance in predicting poorer PFS and OS (Table S3). No statistically significant correlation was found between lesion size and treatment cohort.

## 4. Discussion

Local ablative therapies are increasingly utilized in the treatment of metastatic disease. At our institution, we observed differences in patient selection for ablative treatment of lung metastases with either RFA or SBRT. For instance, almost all sarcoma patients were treated with RFA (96%), as were the majority of colorectal patients (75%). For such patients, substantial bodies of supporting evidence exist for both RFA [7,8,9,10,11,12] and SBRT [6,13,14,15,16,17,18,19,20]. Conversely, all melanoma patients at our institution were treated with SBRT. There is limited evidence specifically reviewing the use of either RFA or SBRT in melanoma patients [21,22]; therefore, it would be justifiable to use either. In our institution, cases of lung metastases where local ablative therapy is considered are discussed in the SBRT multi-disciplinary meeting, where clinical oncologists and interventional radiologists both attend, allowing a balanced discussion of treatment modalities. A consensus opinion is obtained prior to treatment being offered to patients.

In the UK, funding may play a significant role. During the periods specified in the current study, the NHS Clinical Commissioning Policy [26] (previously the Commissioning Through Evaluation Programme) and subsequent NHS England commissioning criteria stipulated that funding for SBRT was permitted for up to a maximum of three separate metastases (metachronous presentation). In patients with synchronous metastases at diagnosis or three or more metastases at any time, SBRT is not a treatment option in the NHS. There are also clinical factors that influence decision-making. (i) Regarding size, smaller lesions, such as those less than 5 mm, can be more technically difficult to treat with SBRT. This is due to difficulties relating to treatment verification or dose build-up in small target volumes [27]. (ii) The potential need for multiple ablative treatments to the lung may favour RFA as the treatment of choice. With the high frequency of lung metastases and recurrences in STS, this may explain why RFA is preferentially used at our institution. During the specified study periods, lung re-irradiation was less common due to uncertainties surrounding the feasibility and safety of retreatment with SBRT or treatment of multiple lesions [28,29], as well as the restrictions imposed by funding. However, as SBRT lung re-irradiation is increasingly adopted [30,31,32], SBRT presents a valid option for local ablative treatment, even in those with prior irradiation.

We observed that more RFA patients received systemic therapy, both prior to and after receiving RFA. This cohort contained fewer patients with oligometastatic disease. This may also reflect the initial decision-making process: that patients predicted to have a high chance of lung recurrence may have been preferentially treated with RFA due to the abovementioned concerns surrounding re-irradiation with SBRT. These patients would have also been more likely to require systemic therapy.

The excellent local control observed in the RFA group may have been confounded by several factors, including tumour size and histological primary. The lesions treated with RFA were smaller overall than those treated with SBRT, with the smallest lesion being 3 mm and the median diameter 11 mm. Larger tumours have been associated with poorer prognosis regardless of treatment modality: poorer local control [29], PFS [7,8,11,20], and OS [8,11,12,18,20]. Multivariable analyses in our series confirmed that larger lesions independently predict poorer rates of LPFS, PFS and OS.

Focusing on histological primary, the majority of the local failures within the SBRT group were with colorectal cancer patients. This correlates with findings from a meta-analysis which found the local control rate of treated colorectal lung metastases to be 60% at three years [33]. Furthermore, colorectal metastases may be more radioresistant than metastases from other primary malignancies. This is supported by the findings from a study exploring radiobiological parameters for lung and liver metastases which looked at over 3700 metastases from 62 studies and found that colorectal metastases were estimated to have a higher alpha/beta ratio [34]. Colorectal lung metastases treated with RFA were associated with better local control than those treated with SBRT in our series. There is increasing evidence that in SBRT, higher BED doses are needed in colorectal metastases to achieve local control, wtih doses similar to those used in melanoma and RCC [35,36,37,38,39]. Lee et al. showed statistically significant differences in local control between colorectal lung metastases receiving BED Gy10 doses of <150 vs. ≥150 Gy [39]. In addition, EGFR and RAS mutations are known to confer radioresistance in colorectal cancers, and some studies have investigated adding tyrosine kinase inhibitor or monoclonal antibody as a radiosensitizer [40,41,42,43,44]. Future studies would benefit from (i) mutation-stratification for local ablative therapies and (ii) the addition of radiosensitizers in metastasis-directed radiotherapy.

The authors acknowledge difficulties with radiological assessment. It can be difficult to differentiate between radiation-induced lung injury and progression, and there may be a tendency to err on the side of caution and diagnose recurrence. We attempted to eliminate this potential for overdiagnosis by independently reviewing all imaging from all patients in both groups with two radiologists, using the High-Risk Factors proposed by Huang and colleagues [25] for those treated with SBRT. PET-CT or histological confirmation were used to confirm local progression in our series. Despite efforts such as these, assessment of local control following SBRT continues to carry uncertainties. A lack of histopathological confirmation at the time of local control failure was a limitation, although this was addressed by dual independent radiological review using the objective criteria detailed in Section 2.2. If re-biopsy is not feasible, then serial imaging should be pursued.

Other limitations of this study include the following: (1) There were differences in characteristics of patients treated with SBRT and RFA. However, we feel this study highlights patient features where one modality may be preferable over the other. (2) Small patient numbers in some tumour subtypes and relatively low numbers of local progression events limited LPFS analysis. (3) Patients were excluded if they did not have at least one follow-up scan following treatment, possibly causing selection bias. The reason for this criterion is that local PFS was the primary objective being assessed, and a minimum of one follow-up scan was therefore deemed essential. Finally, as with any retrospective studies, the data were subject to information and recall bias.

Our study highlighted differences in patient selection for RFA or SBRT in the treatment of lung metastases at our institution, accepting the differences in the two cohorts. For example, SBRT was primarily used in the oligometastatic setting. While SBRT was associated with better overall PFS in Kaplan–Meier analysis, this effect was no longer maintained in multivariable analysis. Another significant finding was that RFA was associated with better local control in colorectal patients, supporting the outcomes from other studies which have suggested greater radioresistance in colorectal cancer. Higher BED doses in SBRT were associated with better local control—again, a finding which supports those from other studies [32,33,34,35,36,37,38,39]. Future research should focus on optimizing patient selection for the different modalities of treatment, defining radiation dose schedules for radiosensitive and radioresistant tumour types, and stratification according to mutation status.

## Figures and Tables

**Table 1:** Baseline characteristics of patients treated with SBRT or RFA between November 2011 and December 2019 (IQR: Interquartile range, * denotes statistical significance).

	SBRT	RFA	*p*-Value
Total number of patients	70	106	*p* = 0.365
Median follow-up (months)	29.7	32.2	*p* = 0.551
Male	44 (63.4%)	53 (50%)	*p* = 0.093
Female	26 (36.6%)	53 (50%)	
Median age (years, IQR)	70.5 (60.0, 78.0)	65 (52.9, 72.6)	*p* = 0.018 *
Oligometastatic disease	64 (91.4%)	20 (20%)	*p* < 0.001 *
ECOG PS ≤ 2	70 (100%)	105 (99%)	*p* = 0.431
Median tumour size (IQR)	18.0 (13.0, 25.5)	11.0 (8, 20)	*p* < 0.001 *
Number of lesions treated per episode (median, range)	1 (1–2)	1 (1–3)	
More than one lesion treated simultaneously	9 (12.9%)	29 (27.4%)	*p* = 0.022 *
Prior systemic therapy	39 (55.7%)	76 (76%)	*p* = 0.005 *
Further systemic therapy	35 (51.4%)	72 (72%)	*p* = 0.003 *

## Data Availability

The original contributions presented in this study are included in the article/Supplementary Material. Further inquiries can be directed to the corresponding authors.

## Supplementary Materials

The following supporting information can be downloaded at: https://www.mdpi.com/article/10.3390/curroncol32060303/s1, Table S1: Local control stratified by primary site and treatment modality; Table S2: Biologically effective dose calculation for patients receiving SBRT; Table S3: Univariable and multivariable Cox-regression analysis.
