# Supplementary material for: Ablative Techniques for Lung Metastases: Patient Selection and Outcomes Following Treatment with Stereotactic Radiotherapy or Radiofrequency Ablation
Source: Curr Oncol. 2025 May 25;32(6):303. doi: 10.3390/curroncol32060303 (PMC12191657; doi:10.3390/curroncol32060303)
Supplement: Supplementary file 1 [file curroncol-32-00303-s001.zip › curroncol-3602788-supplementary.pdf]

Table S1. Local control stratified by primary site and treatment modality. (Primary sites with combined numbers treated <=3 were not shown. RCC Renal cell carcinoma; STS Soft tissue sarcoma.)

**Primary site**	**RFA (total n, (control%))**	**SBRT (total n, (control%))**
Breast	2 (50%)	2 (50%)
Colon	39 (89.7%)	14 (64.2%)
Endometrium	4 (75%)	10 (80%)
Melanoma	0 (N/A)	13 (100%)
Oesophagus	2 (100%)	3 (66.7%)
Prostate	0 (N/A)	3 (100%)
STS	33 (78.8%)	1 (100%)
RCC	3 (67%)	10 (60%)
Rectal	53 (96.2%)	7 (71.4%)
Small bowel	4 (75%)	0 (N/A)

Table S2. Biologically-effective dose calculation for patients receiving SBRT.

Dose	Fractions	Histology	Local Control (1= Local control, 2= Local recurrence)	BED*	*Alpha/beta ratios
45	3	Adenoid cystic carcinoma	1	382	Prostate: 1.5
60	8	Breast ca	1	172.5	Adenoid cystic: 2
50	8	Breast cancer	2	128.1	Melanoma: 2.5
55	5	Cervical SCC	1	115	RCC, Thyroid papillary: 3
54	3	Cervix adenocarcinoma	1	151	Breast, sarcoma: 4
60	8	Colorectal ca	1	105	Colorectal, oesophageal, cervical, endometrial, head&neck, pancreatic, penile: 10
60	8	Colorectal ca	2	105	
60	8	Colorectal ca	1	105	
60	8	Colorectal ca	1	105	
55	5	Colorectal ca	2	115	
60	8	Colorectal ca	1	105	
60	8	Colorectal ca	1	105	
55	5	Colorectal ca	1	115	
54	3	Colorectal ca	2	151	
55	5	Colorectal ca	1	115	
45	3	Colorectal ca	2	112	
54	3	Colorectal ca	2	151	
50	5	Colorectal ca	2	100	
60	8	Colorectal ca	1	105	
60	3	Colorectal ca	2	180	
50	8	Colorectal ca	1	81	

44	8	Colorectal ca	1	68	
50	5	Colorectal ca	1	100	
55	5	Colorectal ca	1	115	
55	5	Colorectal ca	1	115	
55	5	Colorectal ca	1	115	
60	8	Endocervical adenocarcinoma	2	105	
50	5	Endocervical adenocarcinoma	1	100	
60	8	Endometrial adenocarcinoma	1	105	
55	5	Endometrial adenocarcinoma	1	115	
54	3	Endometrial adenocarcinoma	1	151	
55	5	Endometrial adenocarcinoma	1	115	
54	3	Endometrial carcinoma	1	151	
60	8	Endometrial carcinoma	2	105	
54	3	Endometrial serous carcinoma	1	151	
55	5	Endometrial serous carcinoma	1	115	
55	5	Head and neck SCC	1	115	
55	5	Melanoma	1	297	
60	8	Melanoma	1	240	
60	8	Melanoma	1	240	
54	3	Melanoma	1	442.8	
54	3	Melanoma	1	442.8	
55	5	Melanoma	1	297	
54	3	Melanoma	1	442.8	
60	3	Melanoma	1	540	
54	3	Melanoma	1	442.8	

54	3	Melanoma	1	442.8	
60	8	Melanoma	1	240	
55	5	Melanoma	1	297	
50	5	Melanoma	1	250	
55	5	Oesophageal adenocarcinoma	1	115	
60	8	Oesophageal adenocarcinoma	1	105	
55	5	Oesophageal adenocarcinoma	2	115	
56	8	Pancreatic adenocarcinoma	2	95	
60	8	Penile carcinoma	2	105	
55	5	Pleomorphic sarcoma	1	206	
60	8	prostate	1	360	
60	8	Prostate adenocarcinoma	1	360	
60	8	Prostate adenocarcinoma	1	360	
55	5	RCC	1	257	
60	8	RCC	1	210	
60	8	RCC	1	210	
55	5	RCC	1	257	
50	5	RCC	1	217	
54	3	RCC	2	378	
54	3	RCC	2	378	
55	5	RCC	2	257	
60	8	RCC	1	210	
60	8	RCC	1	210	
54	3	Thyroid papillary carcinoma	1	378	
60	8	Thyroid papillary carcinoma	1	210	

Table S3. Univariable and multivariable Cox-regression analysis.

Univariate analysis

**Covariate**		Adjusted HR (95% CI)	p-Value
Treatment modality: RFA vs SBRT	Local PFS	0.57 (0.28,1.14)	0.113
	PFS	1.57 (1.11,2.21)	0.01*
	OS	1.15 (0.71,1.86)	0.582
Lesion size: >20mm vs <20mm	Local PFS	3.32 (1.61,6.86)	0.001*
	PFS	1.57 (1.11,2.23)	0.011*
	OS	2.17 (1.35,3.50)	0.001*
Lesions treated simultaneously: >1 vs 1	Local PFS	1.17 (0.50,2.73)	0.722
	PFS	0.99 (0.67-1.47)	0.956
	OS	0.90 (0.50,1.61)	0.715
Previous SACT: no vs yes	Local PFS	1.29 (0.61,2.70)	0.506
	PFS	0.90 (0.62,1.29)	0.557
	OS	0.63 (0.37,1.08)	0.091
Subsequent SACT: no vs yes	Local PFS	0.61 (0.28,1.36)	0.228
	PFS	0.46 (0.31,0.67)	<0.001*
	OS	0.78 (0.46,1.31)	0.343
Oligometastatic: no vs yes	Local PFS	0.94 (0.32,2.75)	0.904
	PFS	0.92 (0.57,1.47)	0.715

	OS	1.39 (0.73,2.66)	0.318
Colorectal primary: no vs yes	Local PFS	0.95 (0.43,2.06)	0.889
	PFS	0.96 (0.68,1.35)	0.799
	OS	1.34 (0.81,2.20)	0.25

Multivariate analysis

**Covariate**		HR (95% CI)	p-Value
Treatment modality: RFA vs SBRT	Local PFS	1.27 (0.46,2.96)	0.738
	PFS	1.31 (0.89,1.93)	0.17
	OS	1.03 (0.61,1.73)	0.914
Lesion size: >20mm vs <20mm	Local PFS	3.28 (1.59,6.78)	0.001*
	PFS	1.47 (1.03,2.11)	0.034*
	OS	2.22 (1.38-3.58)	0.001*
Subsequent SACT: no vs yes	PFS	0.48 (0.32,0.70)	<0.001*
Colorectal primary: no vs yes	Local PFS	2.42 (0.86,6.87)	0.096
	PFS	1.07 (0.75,1.54)	0.694
	OS	1.39 (0.85,2.26)	0.189
Colorectal primary treated with RFA vs SBRT	Local PFS	0.21 (0.05,0.90)	0.035*
